# Supplementary material for: Chronic Stress Induces Type 2b Skeletal Muscle Atrophy via the Inhibition of mTORC1 Signaling in Mice
Source: Med Sci (Basel). 2023 Feb 10;11(1):19. doi: 10.3390/medsci11010019 (PMC9944114; doi:10.3390/medsci11010019)
Supplement: Supplementary file 1 [file medsci-11-00019-s001.zip › medsci-2188736-supplementary.pdf]

A

glucocorticoid receptor

p-S6

p-4E-BP1

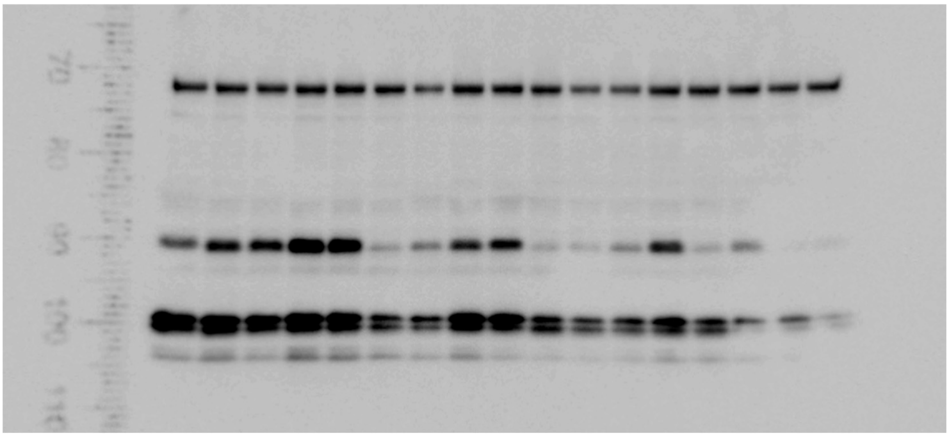

B

tubulin

GAPDH

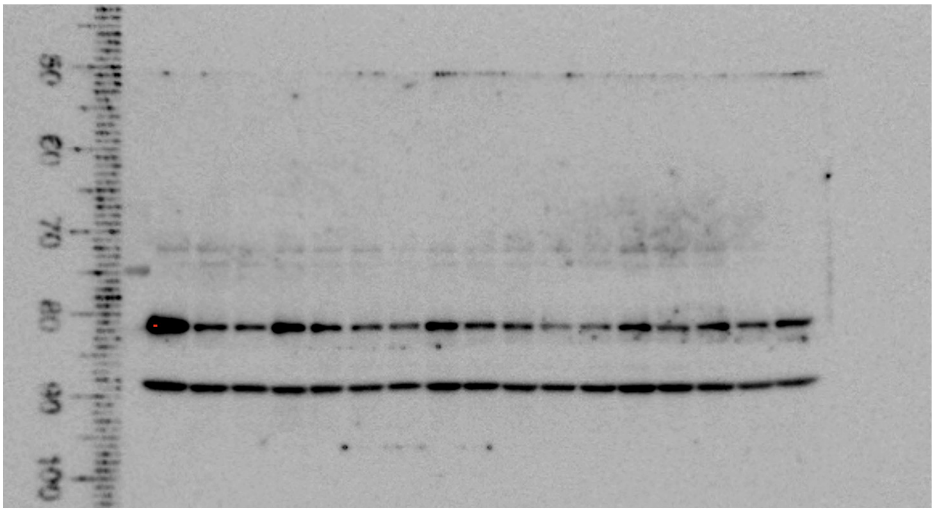

Figure S1. Western blot analysis in all mice. These are supplemental data for Figure 4B. (A) Phosphorylation of S6 and 4E-BP1. (B) Internal control. Lane 2-9: control group and Lane 10 – 17: WRS group. We used lane 8-11 for Figure 4B.
